# Oral health knowledge and practices of people living with type 2 diabetes in three health facilities in the Dschang Health District, West Region, Cameroon

**DOI:** 10.11604/pamj.2025.52.184.44753

**Published:** 2025-12-24

**Authors:** Christian Deube Ngako, Sylvain Raoul Simeni Njonnou, Clarisse Mapa-Tassou, Herna Stella Chimy Tchounchui, Fernando Kemta Lekpa, Faustin Atemkeng Tsatedem, Jerôme Ateudjieu, Siméon Pierre Choukem

**Affiliations:** 1 Department of Epidemiology and Public Health, Faculty of Medicine and Pharmaceutical Sciences, University of Dschang, Dschang, Cameroon,; 2 Department of Internal Medicine and Specialities, Faculty of Medicine and Pharmaceutical Sciences, University of Dschang, Dschang, Cameroon,; 3 Dschang Regional Annex Hospital, Dschang, Cameroon,; 4 Ministry of Public Health, Yaounde, Cameroon,; 5 Douala General Hospital, Douala, Cameroon,; 6 Department of Surgery and Specialities, Faculty of Medicine and Pharmaceutical Sciences, University of Dschang, Dschang, Cameroon,; 7 Deido District Hospital, Douala, Cameroon,; 8 M.A. SANTÉ, Yaounde, Cameroon,; 9 Health and Human Development (2HD) Research Network, Douala, Cameroon

**Keywords:** Type 2 diabetes, knowledge, practices, oral health, Cameroon

## Abstract

**Introduction:**

people with type 2 diabetes mellitus (T2DM) are at increased risk of periodontal disease (POD), which gets complicated with diabetes. Good oral hygiene and regular dental check-ups are recommended to prevent oral health problems, including POD. This study aimed to assess the oral health knowledge and practices of people with T2DM in three health facilities of the Dschang Health District (West Region, Cameroon).

**Methods:**

we conducted a cross-sectional study over five months targeting people living with T2DM, followed up in three health facilities of the Dschang Health District, and who agreed to participate. Data were collected using a face-to-face questionnaire. The variables studied were sociodemographic and clinical characteristics, knowledge of the relationship between diabetes and POD, frequency of dental visits and oral hygiene practices. Knowledge and practices were considered good for a response score > 75% and poor for a score < 25%. Intermediate scores were considered average and insufficient. Bivariate and multivariate analyses were performed with a significance level (p <0.05).

**Results:**

we recruited 217 (115 women) participants living with T2DM. The median age of participants was 62 (55-68.5) years. More than half (57.6%) had poorly controlled T2DM. Overall, two-thirds (66.8%) of participants had insufficient knowledge, while 12.4% only had good knowledge. More than a third of participants (39.6%) had poor practices and only 13.8% had good practices. The factors associated with good knowledge were secondary education (ORa = 2.819; 95% CI [1.001-7.943]; p=0.049) and retired status (ORa = 4.269; 95% CI [1.827-9.971]; p=0.001). Having a well-controlled T2DM was associated with good practices (ORa = 2.284; 95% CI [1.032-5.055]; p=0.042).

**Conclusion:**

most people living with T2DM have insufficient knowledge of oral health, poor oral health practices and fewer visits to the dentist. Secondary education and retired status were associated with good knowledge, while only well-controlled T2DM was associated with good practices. It is therefore vital to incorporate oral health education into their follow-up and education.

## Introduction

Diabetes is one of the major chronic conditions that occurs when the body cannot produce enough insulin or cannot effectively use the insulin it does produce. It affected approximately 537 million adults aged 20-79 years worldwide in 2021, both in developing and developed countries. With current trends, it has been projected that about 784 million people will have diabetes in 2045 [1]. Diabetes also imposes a substantial economic burden on countries, health systems, people with diabetes, and their families; at least USD 966 billion was spent on healthcare expenditure in 2021 globally [1]. Africa is expected to experience the greatest increase in the number of cases of diabetes in the world, rising from 24 million cases in 2021 to 55 million in 2045 [1]. Type 2 diabetes is the most common form, accounting for more than 90% of people living with diabetes, and is currently considered one of the world's health emergencies [2]. According to the International Diabetes Federation (IDF), the prevalence of type 2 diabetes in Cameroon was 4.8% [1]. A meta-analysis done in 2018 found a prevalence of 5.8% in Cameroon [3].

Diabetes can lead to several complications related to different organ systems, including the eyes, kidneys, nerves, heart, and blood vessels [2]. Although not often discussed in diabetes care, people living with diabetes are also at increased risk of developing oral health problems, particularly periodontal disease (POD) [4]. This includes both gingivitis and periodontitis. Gingivitis is the mildest form of POD in which inflammation is confined to the gums and can be reversed with effective oral hygiene, whereas periodontitis is the advanced stage in which inflammation extends deep into the tissues and causes irreversible destruction of the supporting tissues of the tooth [5]. Its clinical signs include loss of clinical attachment, bone lysis, periodontal pockets and gingival inflammation [5]. Left untreated, it leads to premature tooth loss. According to the World Health Organization (WHO), periodontitis is one of the main causes of tooth loss in adults [6]. Essama *et al*. in a 2013 study conducted in Cameroon, reported that all people living with T2DM had POD [7]. Another study conducted in 2020 at Douala General Hospital showed a 52% prevalence of periodontitis in people living with diabetes [8]. It has been established that diabetes and periodontitis are linked. Chronic hyperglycemia affects periodontal outcomes and periodontitis also increases the blood glucose levels [9]. The evidence for an association between diabetes and periodontitis is strongest for T2DM [10]. People with T2DM are 2 to 3 times more likely to develop periodontitis than those without diabetes [11]. Current evidence from interventional studies suggests that periodontal treatment may improve glycemia [12].

Given the impact of POD on the health of people living with T2DM and the benefits of good oral health practice in minimising the risk of POD, it is important to ensure that people living with T2DM are motivated to adopt good oral hygiene behaviours and that they benefit from risk assessment and referral to a dentist. Adequate oral health knowledge is positively associated with good behaviours such as increased frequency of tooth brushing and dental visits and good periodontal health [13]. Studies by several researchers in other settings have shown that most people living with diabetes have poor oral health knowledge, poor oral health attitudes and fewer dental visits [14-16]. They rarely receive oral health education and guidance from their healthcare providers [14]. Although few studies in our context have assessed the oral health status of people living with diabetes [7,17], we note that data regarding oral health knowledge and practices in people living with T2DM are almost non-existent. This study aimed to assess the oral health knowledge and practices of people living with T2DM in three health facilities in the Dschang Health District and identify the factors associated with good oral health knowledge and practices. Furthermore, we aimed to assess the impact of sociodemographic and diabetes-related characteristics on good oral health knowledge and practices.

## Methods

**Study design and setting:** we conducted a cross-sectional study targeting people living with T2DM followed up in three health facilities of the Dschang Health District, namely the Dschang Regional Annex Hospital (DRAH), the Saint Vincent de Paul Hospital (SVDPH) and the Batsingla Sisters Hospital (BSH). These three health facilities play a central role in the provision of quality health care for the population of the Dschang Health District (in the Menoua subdivision, West Region, Cameroon), which justifies our choice. The DRAH is a third-category public health facility located in the Dschang district (45 Km from Bafoussam, the capital of the West Region), opposite the University of Dschang. Outpatient consultations and follow-ups of patients with diabetes are carried out in the Internal Medicine department by an endocrinologist and an internist. The SVDPH and the BSH are private religious health facilities where people with diabetes are also followed up in the Dschang Health District.

**Study population:** our study population consisted of all outpatients living with T2DM coming for consultation or follow-up to the Diabetes Unit and Internal Medicine department of these three health facilities, from 1^st^ February to 30^th^ June 2023 (5 months).

**Sample selection**

**Minimum sample size calculation:** it was calculated using the national prevalence of diabetes in 2021 in Cameroon (4.8%) [1], with a margin of error of 5% (Zα=1.96). The minimum size was 70 participants.

**Inclusion and exclusion criteria:** all people with T2DM aged 21 years or older who were approached and gave informed consent during the study period were included. All patients living with T2DM with a diagnosis of less than three months and pregnant women were excluded from the study.

### Procedures

**Administrative and ethical authorisations:** administrative authorisations were obtained from the directorate of the three health facilities. Before data collection, the study proposal was reviewed and approved by the West Regional Ethics Committee, Cameroon (N°312/29/2023/CE/CRERSH-OU/VP).

**Recruitment of people with type 2 diabetes:** people with T2DM were recruited consecutively among those consulting the Internal Medicine Department of the Dschang Regional Annex Hospital, the Batsengla Sisters Hospital, and the Saint Vincent de Paul Hospital in Dschang over the period February to June 2023. The study was therefore explained and for those agreeing to participate, informed consent was collected.

**Data collection:** verbal informed consent was obtained from these participants following an explanation of the study's purpose. Therefore, a pre-designed and pre-tested questionnaire was administered by the principal investigator in a face-to-face interview lasting 10 minutes on average. The questionnaire collected the following data: i) socio-demographic characteristics (age, sex, marital status, level of education, monthly income, area of residence and profession); ii) knowledge (6 questions); iii) practices (14 questions). We also used patients' medical records to collect information on diabetes (duration of diagnosis, control, treatment, complications) and co-morbidities. The aim was to collect data such as socio-demographic and clinical characteristics, knowledge of the relationship between diabetes and POD, oral hygiene practices (frequency, timing, means, method, and duration of tooth brushing) and frequency of dental visits.

### Operational words

***People living with T2DM:*** any person with a documented diagnosis of T2DM or taking T2DM treatment.

***Good diabetes control:*** glycated haemoglobin (HbA1C) less than 7% during the 3 months before the study.

***Diabetes diagnosis duration:*** was recent if < 5 years and long if ≥5 years.

**Documented health problems caused by chronic hyperglycemia such as heart disease, stroke, retinopathy, nephropathy, neuropathy, infections, and foot ulcers**

***Hypertension:*** participant with blood pressure ≥140/90 mmHg or taking any antihypertensive treatment.

***Body weight status:*** was defined according to the body mass index (BMI) as follows: normal weight (18.50 ≤ BMI ≤ 24.99 Kg/m^2^); overweight (25 ≤ BMI ≤ 29.99 Kg/m^2^), obese (BMI ≥ 30 Kg/m^2^).

***Physical activity:*** was defined as the presence of at least 3 walking episodes of 45 min in a week.

***Monthly income:*** low (< USD 86.3), medium (USD 86.3 and 258.9) and high (> USD 258.9).

***Tobacco consumption:*** subject declaring smoking or having stopped smoking tobacco less than 6 months ago.

**Statistical analysis:** data were collected by Census and Survey Processing (CsPro) 7.5 and analyzed by Statistical Package for Social Sciences (SPSS) 23 software. Results were presented in tables and figures. Qualitative variables were expressed as proportions with a 95% confidence interval. Quantitative (continuous) variables were expressed as median (interquartile range). To assess knowledge, we used the following classification: poor (score < 25% of points); insufficient (score < [25-50% [ of points); average (score = [50-75%] of points) and good (score > 75% of points). Practices were poor (score <25% of points), insufficient (score < [25-50% [ of points), average (score = [50-75%] of points) and good (score >75% of points). The Chi-square test and Fisher's exact test were used to compare proportions in bivariate analysis. The comparison groups were: good knowledge vs other levels of knowledge; adequate (good) practices vs other levels of practices. Multivariate analysis with calculation of adjusted odds ratios and 95% confidence intervals was used to determine the factors associated with good knowledge and good practices. We included in the multivariate model the variables with p<0.05 in the bivariate analysis. Differences were considered statistically significant for values of p<0.05.

**Ethical considerations:** administrative authorisations were obtained from the directorate of the three health facilities. Before data collection, the study proposal was reviewed and approved by the West Regional Ethics Committee, Cameroon (N°312/29/2023/CE/CRERSH-OU/VP). Informed consent was obtained from each participant. The questionnaire adhered to the rules of anonymity, and the information collected during our study was kept confidential. The data and results were used for purely scientific purposes. This research was conducted in accordance with the Helsinki principles.

## Results

### Main data

**Socio-demographic characteristics:** we included 217 (115 women) participants. The median age was 62 (55 - 68.5) years, with extremes ranging from 30 to 94 years. Most of the participants (60.4%) had a secondary education. The majority (80.2%) were married, with almost a third (32.7%) not working and just over a quarter (28.1%) retired. More than a third (37.8%) had a monthly income of less than USD 86.3. Another third (33.6%) had a monthly income of more than USD 258.9 (Table 1).

**Table 1:** distribution of the socio-demographic features of participants with T2DM in Dschang Health District (N=217)

Socio-demographiccharacteristics	Results (N=217)
**Median age [Interquartile range] (years)**	62 [55 -68,5]
**Sex**	
Male	102 (47.0)
Female	115 (53.0)
**Age groups (years)**	
< 45	6 (2.8)
[45-65[	123 (56.7)
[65-85[	87 (40.1)
≥ 85	1 (0.5)
**Level of education**	
No education	20 (9.2)
Primary	41 (18.9)
Secondary	131 (60.4)
University	25 (11.5)
**Marital status**	
Single	2 (0.9)
Married	174 (80.2)
Widowed	1 (0.5)
Divorced	40 (18.4)
**Profession**	
Not working/Homemaker	71 (32.7)
Self-employed	54 (24.9)
Employed (public/private)	31 (14.3)
Retired	61 (28.1)
**Monthly income (USD)**	
< 86.3	82 (37.8)
[86.3 - 258.9[	62 (28.6)
≥ 258.9	73 (33.6)

Monthly income: low: Monthly income of less than USD 86.3; Medium: income between USD 86.3 and USD 258.9; and high: income above USD 258.9. *T2DM: Type 2 Diabetes Mellitus

**Clinical characteristics:** more than half (57.6%) of the participants had poor glycemic control. Nearly two-thirds (63.6%) had been diagnosed with diabetes for more than five (5) years. In addition, 37.3% had irregular follow-up. Hypertension was the most common comorbidity (39.6%). More than half the participants (51.6%) had at least one complication of T2DM. More than half of the participants (50.2%) said they had used herbal medicine. It should also be noted that 70.5% were compliant with their treatment (Table 2).

**Table 2:** distribution of the clinical features of participants with T2DM in Dschang Health District (N=217)

Clinicalcharacteristics	Results (N=217)
**Glycemic control**	
Poor (HbA1c ≥ 7%)	125 (57.6)
Good (HbA1c < 7%)	92 (42.4)
**Diagnostic duration T2DM (years)**	
≤ 5	79 (36.4)
> 5	138 (63.6)
**Regular follow-up (at least one visit per trimester)**	
Yes	136 (62.7)
No	81 (37.3)
**Hypertension**	
Yes	86 (39.6)
No	131 (60.4)
**T2DM complications**	
Yes	112 (51.6)
No	105(48.4)
**Treatment adherence**	
Yes	153 (70.5)
No	64 (29.5)
**Phytotherapy consumption**	
Yes	109 (50.2)
No	108 (49.8)

**Figure 1:**
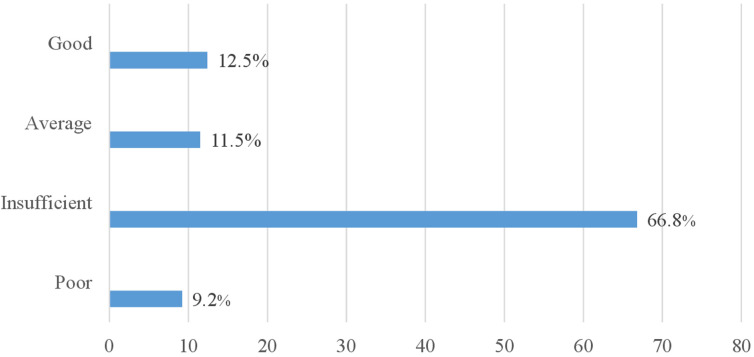
distribution of population according to the level of knowledge

**Oral health knowledge:** only 18% of people living with T2DM were aware of the existence of a relationship between diabetes and POD. For 15.7% of them, the dentist had a role to play in monitoring diabetes. Participants were much more aware of dental caries (87.1%) than periodontitis (6.9%). Overall, two-thirds (66.8%) of participants had insufficient knowledge and only 12.5% had good knowledge (Table 3 and Figure 1).

**Table 3:** T2DM participants' knowledge about periodontal diseases in the Dschang Health district (N=217)

Knowledge about oral health (N=217)	n (%)	
	**Correct answer**	**Wrong answers**
Have you ever heard of periodontal disease?	15 (6.9)	202 (93.1)
Knows periodontal disease?	7 (3.2)	210 (96.8)
Know how to prevent periodontal disease?	15 (6.9)	202 (93.1)
Is there a link between diabetes and periodontal disease?	39 (18.0)	178 (82.0)
Knows that diabetes increases the risk of periodontal disease?	37 (17.1)	180 (82.9)
Have you ever heard of tooth decay?	189 (87.1)	28 (12.9)
Knows tooth decay?	54 (24.9)	163 (75.1)
Know how to prevent tooth decay?	153 (70.5)	64 (29.5)
Does the dentist have a role to play in monitoring diabetes?	34 (15.7)	183 (84.3)

**Figure 2:**
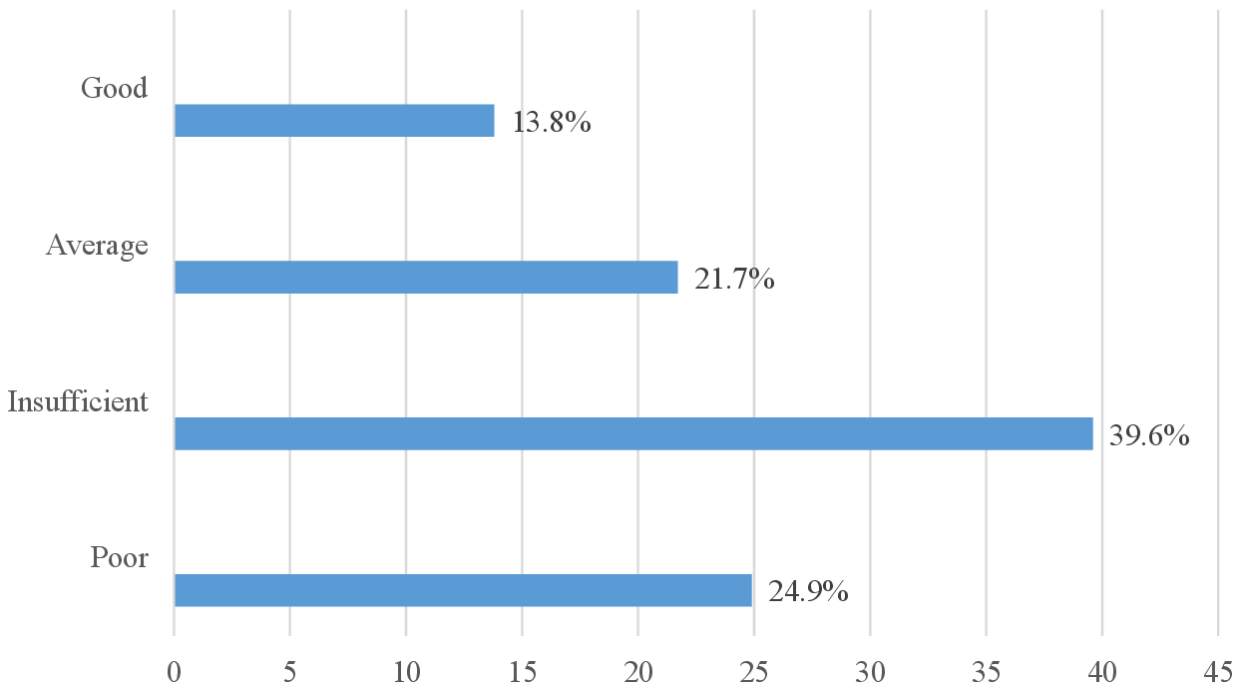
distribution of population according to the level of practices

**Dental practices:** more than half (51.2%) of the participants had never had an oral health check-up. However, nearly a third (33.2%) had already had one, but more than two years previously. Nearly two-thirds (63.1%) of people with T2DM cleaned their teeth once a day, in the morning before meals (58.1%) and using a toothbrush and toothpaste (68.2%). However, just over a quarter (25.3%) used a toothbrush without additives. Less than half of the participants (44.7%) had a good brushing method, and more than three-quarters (77.4%) brushed for more than three minutes. More than half (55.6%) of participants said they change their toothbrush when it becomes worn (or lost). Overall, more than a third of participants (39.6%) had inadequate practices and only 13.8% had good practices (Table 4 and Figure 2).

**Table 4:** oral health practices of people living with T2DM in the Dschang Health District (N=217)

Oral health practices	Results (N=217)
**How often do you visit the dentist each year?**	
Once	12 (5.5)
Twice	0
In case of a problem	94 (43.3)
Never	111 (51.2)
**When was your last dental appointment?**	
One year	15 (6.9)
Two years	19 (8.8)
More than two years	72 (33.2)
Never	111 (51.2)
**Do you brush your teeth?**	
Yes	217 (100)
No	0
**How many times a day do you brush your teeth?**	
Once	137 (63.1)
Twice	80 (36.9)
When do you brush your teeth?	
In the morning before meals	126 (58.1)
In the evening at bedtime	11 (5.1)
Morning before meal and evening at bedtime	24 (11.1)
Morning after meal and evening at bedtime	56 (25.8)
**What do you use to brush your teeth?**	
Toothbrush and toothpaste	148 (68.2)
Toothbrush only	55 (25.3)
Toothbrush and ash/charcoal	6 (2.7)
Toothbrush and salt	3 (1.4)
Toothbrush and bicarbonate	3 (1.4)
Toothbrush and honey	1 (0.5)
Plant stem	1 (0.5)
**What brushing technique do you use?**	
Good	97 (44.7)
Bad	120 (55.3)
**How many minutes do you brush your teeth?**	
< 3	49 (22.6)
≥ 3	168 (77.4)
**How often do you change your toothbrush?**	
When it's worn out / lost	120 (55.6)
After 3 months of use	70 (32.4)
> 3 months	26 (12.0)

**Factors associated with good knowledge and practices:** after bivariate analysis, secondary education (OR = 3.270; 95% CI [1.188-9.002]; p=0.017) and retired status (OR = 4.687; 95% CI [2.029-10.828]; p<0.001) were associated with good knowledge. After multivariate analysis, this association between secondary education (ORa = 2.819; 95% CI [1.001-7.943]; p=0.049), retired occupation (ORa = 4.269; 95% CI [1.827-9.971]; p=0.001) and good knowledge was still present (Table 5). Well-controlled T2DM (OR = 2.291; 95% CI [1.043-5.032]; p=0.036) and non-use of herbal medicine (OR = 2.250; 95% CI [1.001-5.066]; p=0.046) were associated with good practice in bivariate analysis. After multivariate analysis, having a well-controlled T2DM (ORa = 2.284; 95% CI [1.032-5.055]; p=0.042) was associated with good practices (Table 6).

**Table 5:** factors associated with good knowledge among people with T2DM living in the Dschang Health District (N=217)

Variables	Terms	Good knowledge n (%)	Other knowledge level n (%)	Crude OR (CI 95%)	p-value	Adjusted OR (CI 95%)	Adjusted p-value
**Age (years)**	< 62	8 (29.6)	91 (47.9)	0.458 (0.191-1.097)	0.075	-	-
	≥ 62	19 (70.4)	99 (52.1)	-		-	-
**Sex**	Male	11 (40.7)	104 (54.7)	0.569 (0.251-1.290)	0.569	-	-
	Female	16 (59.3)	86 (45.3)	-		-	-
**Level of education**	No education	2 (7.4)	39 (20.5)	0.310 (0.070-1.364)	0.103	-	-
	Primary	0	20 (10.5)	Not applicable	0.145	-	-
	Secondary	22 (81.5)	109 (57.4)	3.270 (1.188-9.002)	0.017	2.819 (1.001-7.943)	0.049
	University	3 (11.1)	22 (11.6)	0.955 (0.265-3.433)	1	-	-
**Profession**	Not working/Homemaker	7 (25.9)	64 (33.7)	0.689 (0.277-1.715)	0.421	-	-
	Self-employed	3 (11.1)	51 (26.8)	0.341 (0.098-1.180)	0.077	-	-
	Employed (public/private)	1 (3.7)	30 (15.8)	0.205 (0.027-1.570)	0.139	-	-
	Retired	16 (59.3)	45 (23.7)	4.687 (2.029-10.828)	<0.001	4.269 (1.827-9.971)	0.001
**Monthly income (USD)**	< 86.3	11 (40.7)	71 (37.4)	1.152 (0.506-2.622)	0.735	-	-
	[86.3 -258.9[	9 (33.3	53 (27.9)	1.292 (0.547-3.056)	0.558	-	-
	≥ 258.9	7 (25.9)	66 (34.7)	0.658 (0.264-1.635)	0.365	-	-
**Glycemic control**	Good (HbA1c < 7%)	12 (44.4)	80 (42.1)	1.100 (0.488-2.477)	0.818	-	-
	Poor (HbA1c ≥ 7%)	15 (55.6)	110 (57.9)	-	-	-	-
**T2DM disease duration**	≤ 5 years	9 (33.3)	70 (36.8)	0.857 (0.365-2.011)	0.723	-	-
	> 5 years	18 (66.7)	120 (63.2)	-	-	-	-
**Regular follow up**	Yes	15 (55.6)	121 (63.7)	0.713 (0.316-1.610)	0.414	-	-
	No	12 (44.4)	69 (36.3)	-	-	-	-
**Hypertension**	Yes	11 (40.7)	75 (39.5)	1.054 (0.464-2.396)	0.900	-	-
	No	16 (59.3)	115 (60.5)	-	-	-	-
**T2DM complications**	Yes	14 (51.9)	98 (51.6)	1.011 (0.451-2.265)	0.979	-	-
	No	13 (48.1)	92 (48.4)			-	-
**Treatment adherence**	Yes	19 (70.4)	134 (70.5)	0.993 (0.410-2.400)	0.987	-	-
	No	8 (29.6)	56 (29.5)			-	-
**Phytotherapy consumption**	Yes	13 (48.1)	96 (50.5)	0.909 (0.406-2.037)	0.817	-	-
	No	14 (51.9)	94 (49.5)			-	-

Only significant predictors were included in multivariable analysis. *T2DM: Type 2 Diabetes Mellitus

**Table 6:** factors associated with good practices among people with T2DM living in the Dschang Health District (N=217)

Variables	Terms	Good practice n (%)	Other practice level n (%)	Crude OR (CI 95%)	p-value	Adjusted OR (CI 95%)	Adjusted p-value
**Age groups**	< 62	13 (43.3)	86 (46.0)	0.898 (0.413-1.954)	0.786	-	-
	≥ 62	17 (56.7)	101 (54.0)	-	-	-	-
**Sex**	Male	15 (50.0)	100 (53.5)	0.870 (0.402-1.881)	0.723	-	-
	Female	15 (50.0)	87 (46.5)	-	-	-	-
**Level of education**	No education	7 (23.3)	34 (18.2)	1.370 (0.544-3.451)	0.503	-	**-**
	Primary	2 (6.7)	18 (9.6)	0.671 (0.147-3.050)	1	-	-
	Secondary	16 (53.3)	115 (61.5)	0.716 (0.330-1.554)	0.396	-	-
	University	5 (16.7)	20 (10.7)	1.670 (0.575-4.851)	0.356	-	-
**Profession**	Not working/Homemaker	8 (26.7)	63 (33.7)	0.716 (0.302-1.698)	0.447	-	-
	Self-employed	9 (30.0)	45 (24.1)	1.352 (0.578-3.163)	0.485	**-**	**-**
	Employed (public/private)	7 (23.3)	24 (12.8)	2.067 (0.801-5.336)	0.157	**-**	**-**
	Retired	6 (20.0)	55 (29.4)	0.600 (0.232-1.549)	0.287	**-**	**-**
**Monthly income (USD)**	< 86.3	9 (30.0)	73 (39.0)	0.669 (0.291-1.541)	0.343	-	**-**
	[86.3 -258.9[	7 (23.3)	55 (29.4)	0.730 (0.296-1.801)	0.494	-	-
	≥ 258.9	14 (46.7)	59 (31.6)	1.898 (0.870-4.144)	0.104	-	-
**Glycemic control**	Good (HbA1c < 7%)	18 (60.0)	74 (39.6)	**2.291 (1.043-5.032)**	**0.036**	**2.284 (1.032-5.055)**	**0.042**
	Poor (HbA1c ≥ 7%)	12 (40.0)	113 (60.4)	**-**	**-**	**-**	**-**
**T2DM disease duration**	≤ 5 years	10 (33.3)	69 (36.9)	0.855 (0.378-1.932)	0.706	-	-
	> 5 years	20 (66.7)	118 (63.1)	-	-	-	-
**Regular follow-up**	Yes	19 (63.3)	117 (62.6)	1.033 (0.465-2.299)	0.936	-	-
	No	11 (36.7)	70 (37.4)	-	-	-	-
**Hypertension**	Yes	13 (43.3)	73 (39.0)	1.194 (0.548-2.604)	0.655	-	-
	No	17 (56.7)	114 (61.0)	-	-	-	-
**T2DM complications**	Yes	14 (46.7)	98 (52.4)	0.795 (0.367-1.721)	0.559	-	-
	No	16 (53.3)	89 (47.6)	-	-	-	-
**Treatment adherence**	Yes	22 (73.3)	131 (70.1)	1.176 (0.494-2.799)	0.715	-	-
	No	8 (26.7)	56 (29.9)	-	-	-	-
**Phytotherapy consumption**	No	20 (66.7)	88 (47.1)	**2.250 (1.001-5.066)**	**0.046**	2.244 (0.989-5.090)	0.053
	Yes	10 (33.3)	99 (52.9)	**-**	**-**	-	-

Only significant predictors were included in multivariable analysis. *T2DM: type 2 Diabetes Mellitus

## Discussion

This study aimed to assess the oral health knowledge and practices of people living with T2DM. Only 12.4% of participants had good knowledge, and two-thirds (66.8%) had insufficient knowledge. Similarly, over a third (39.6%) had poor oral health practices, and only 13.8% had good oral health practices. Factors associated with good knowledge were secondary education and being retired. Having a well-controlled T2DM was associated with good practices.

The present study showed that two-thirds (66.8%) of the sample had insufficient knowledge of oral health. This finding is in line with Poudel *et al*. in 2018, who reported in a systematic review that people living with diabetes have insufficient knowledge about oral health [14]. Indeed, we found that only 18% were aware of a relationship between diabetes and POD. Low proportions have also been described in the literature. Yaser *et al*. reported 17.61% in 2022, while in Nigeria, Nathan *et al*. found 10.3% [16] and Onigbinde *et al*. 30.3% in 2017 [15,16,18]. These results could be justified by the fact that people living with T2DM are not sufficiently informed on the subject. Very few receive information on oral health from the healthcare staff responsible for their care. However, some studies have shown that people who are better informed or have a good knowledge of the link between diabetes and POD are more likely to adopt good oral health behaviours [14,19]. It is therefore important to include oral health education in the education of people living with T2DM for successful overall management. For a minority (15.7%) of our respondents, the dentist has a role to play in monitoring diabetes. This result is lower than those reported by Yaser *et al*. (20.47%) and Onigbinde and al. (38.2%) [15,16]. This result may be explained by the low level of knowledge among participants people about their increased risk of oral problems. However, this result could also reflect the low involvement of dentists in the team responsible for monitoring people living with diabetes. The literature reports that the involvement of dentists in multidisciplinary teams has been shown to have a positive impact in other areas such as antenatal care. For example, the Midwifery Initiated Oral Health (MIOH) program in Australia, in which dentists and midwives work together, has demonstrated a significant improvement in oral health knowledge, midwives' confidence in promoting oral health and the oral health status of pregnant women [20,21]. Only 6.9% of participants reported ever having heard of periodontitis. This result further indicates that periodontitis is not well-known in the diabetic population. According to the literature, people with T2DM are 2 to 3 times more likely to develop periodontitis than those without diabetes [11]. According to the study by Onigbinde *et al*., people with diabetes were more aware of the various systemic complications of diabetes than of the risk of oral complications [15]. There is therefore, an urgent need to devote more attention to educating diabetic people about their risk of periodontitis and the complications that could arise from it.

Content analysis of practices showed that over a third (39.6%) of the sample had poor oral health practices. This is in agreement with Poudel *et al*. in 2018, who found that people living with diabetes had poor compliance with oral hygiene and dental visit behaviours [14]. More than half (51.2%) of the participants had never had an oral health check-up. In addition, 33.2% had not had a dental check-up for more than two years. Similarly, Essama *et al*. in Cameroon found that 53.7% of people with diabetes had never consulted a dentist [7]. Onigbinde *et al*. reported similar proportions (49.3% and 34.5%) [15]. Several factors could explain our results. Firstly, the respondents' low level of knowledge about their increased risk of oral problems and the need for regular dental check-ups. One study showed that participants who had a good knowledge of the link between diabetes and periodontal disease visited the dentist regularly [22]. People with diabetes therefore, need education on the importance of routine dental visits. Secondly, we can mention the fact that the staff in charge of monitoring these people refer very few of them for a routine oral health consultation. According to Haghdoost *et al*. in 2022, only 8.3% of people with diabetes were referred to the dentist by their diabetes specialist [23]. Kirti *et al*. reported that less than half (32.8%) of people with diabetes had been instructed by their doctor to see a dentist [24]. Lastly, the cost of dental care is often cited as a major obstacle by people suffering from chronic diseases, especially in the absence of universal health coverage capable of bearing this burden [25]. More than a third of our participants had a monthly income of less than USD 86.3. Consequently, the feasibility of setting up an affordable and accessible care system for people living with diabetes must also be explored. In contrast to our study, some authors have found much higher proportions of visits to the dentist, particularly in the United States (86.7%), the United Kingdom (85.2%) and Iran (83%), which have specific programs for people with T2DM [26-28].

This study shows that participants' attitudes towards maintaining good oral health need to be improved, as only 36.9% cleaned their teeth twice compared with 63.1% who cleaned only once. This indicates that brushing more often than once a day was not a common practice among our participants. Education on this aspect is urgently needed. Similar results were reported by Onigbinde *et al*. (36.8% and 63.2%) and Haghdoost *et al*. (35% and 65%) [15,23]. The interventional study by Malekmahmoodi *et al*. in 2020 confirmed the optimal efficacy of educational interventions through training, and active and continuous follow-up, thereby improving oral hygiene behaviours in people living with T2DM. Oral hygiene performance in the intervention group increased significantly from 2.16 ± 0.71 to 3.25 ± 0.49 (three months after education) [29]. One positive point to note is the use of toothbrushes by the majority of participants. More than two-thirds (68.2%) used a toothpaste. Toothbrushes and toothpaste are the most widespread and universally accepted means of oral hygiene for maintaining good oral health. However, it is important to note that 25.3% used a toothbrush without toothpaste. The reason given by these people was the fear of increasing their blood sugar levels. This is somewhat paradoxical, since toothpastes should not contain absorbable sugar and therefore should not be able to raise the blood sugar levels of people living with diabetes. It would therefore be necessary, in our context, to investigate the effects of the locally manufactured toothpastes on the salivary and blood components (glucose and pH) of people living with diabetes mellitus. Similarly, we note archaic procedures such as the use of ash, charcoal, salt and plant stems, which reflect either a lack of education or a fear of raising blood glucose levels.

We found an association between secondary education and good knowledge. This result is similar to that of Bahammam *et al*. in 2015, who reported an association between the level of study and better knowledge about oral health [30]. These results could be justified by the fact that the higher the level of education, the higher the susceptibility to a better level of knowledge. Having well-controlled diabetes was associated with good practices. This result could be explained by the fact that well-controlled participants are more exposed to healthcare personnel and therefore receive more information. Al Habashneh *et al*. found that a healthier lifestyle was associated with a greater likelihood of knowing the link between diabetes and oral health in people with diabetes [31].

## Conclusion

This study aimed to assess the oral health knowledge and practices of people living with T2DM. Women were the most represented. Most participants had a secondary education. More than a third had a monthly income of less than USD 86.3. More than half had poor glycemic control. A minority of participants had good knowledge, and two-thirds had insufficient knowledge. More than a third had poor practices, and a minority had good practices. Factors associated with good knowledge were secondary education and being retired. Having a well-controlled T2DM was associated with good practices. These results highlight the need to make more oral health information available to people living with T2DM.

### What is known about this topic

- People with T2DM have a high risk of developing oral diseases, which also negatively impacts the diabetes course and control;
- Good oral hygiene and regular dental follow-up help to control diabetes.

### What this study adds

- People living with T2DM have insufficient knowledge of oral health and poor oral health practices;
- Secondary education and retired status were associated with good knowledge, while only well-controlled T2DM was associated with good practices.
